# Protocol for generating reproducible miniaturized controlled midbrain organoids

**DOI:** 10.1016/j.xpro.2023.102451

**Published:** 2023-07-21

**Authors:** Muwan Chen, Jonathan Christos Niclis, Mark Denham

**Affiliations:** 1 Danish Research Institute of Translational Neuroscience (DANDRITE), Nordic EMBL Partnership for Molecular Medicine, Aarhus University, 8000C Aarhus, Denmark; 2 Department of Biomedicine, Aarhus University, 8000C Aarhus, Denmark; 3 Cell Therapy R&D, Novo Nordisk, 2760 Måløv, Denmark

**Keywords:** Neuroscience, Organoids

## Abstract

Here, we present a protocol for generating miniaturized controlled midbrain organoids (MiCOs) of reproducible size and cellular composition, without a necrotic center. We describe steps for maintaining and passaging human pluripotent stem cells, generating MiCOs using AggreWell^TM^400, and maintaining them in an EB-Disk^360^on an orbital shaker, eliminating the need for Matrigel or a spinner flask and preventing organoid fusion. We then detail organoid collection for different endpoint analysis. This protocol is suitable for compound screening and disease modeling studies.

## Highlights

• Generation of miniaturized midbrain organoids (MiCOs) devoid of a necrotic center
• Cost-effective maintenance of 360 organoids in one well of a 6-well plate
• Protocols for MiCO collection and analysis
• Suitable for high-throughput compound screening and disease modeling studies

## Abstract (editor highlights)

Publisher’s note: Undertaking any experimental protocol requires adherence to local institutional guidelines for laboratory safety and ethics.

## Graphical abstract

**Figure:**
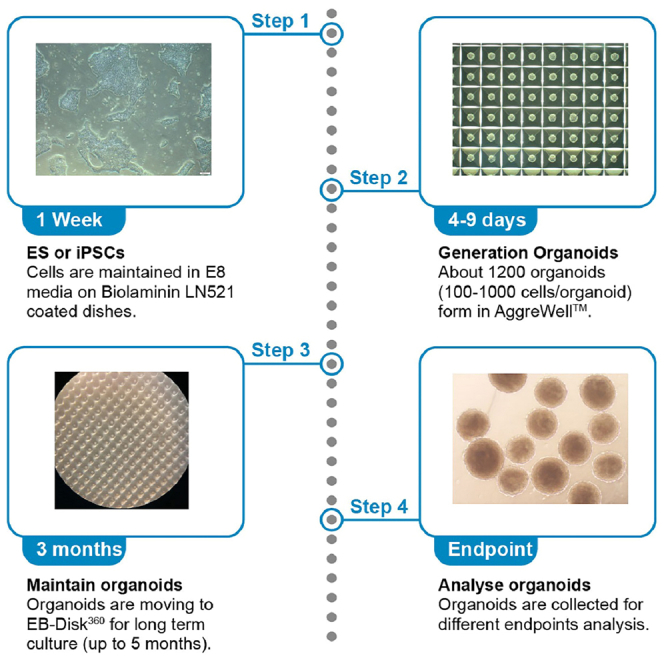

## Before you begin

This protocol is focused on generating reproducible MiCOs without a necrotic center.

We use this protocol to generate ventral midbrain (vmDA) MiCOs. We describe the specific steps for the generation of MiCOs starting directly from human pluripotent stem cells (hPSCs) with the H9 line. However, we also used this protocol to generate MiCOs starting from the neural stem cell stage from widely used 2D protocols. The initial cell number per MiCO needs to be titrated.

***Alternatives:*** We have also used this protocol with an induced pluripotent stem cell line GBA-002-C3 (DANi-002C)^1^ and two genetically modified stem cell line generated in our lab.

***Note:*** When using different stem cell lines for midbrain dopaminergic differentiation, careful titration of the Wnt-agnoist CHIR99021 is necessary. For the H9 cells in our lab, we determined that 0.6 μM CHIR99021 is the optimal condition for dopaminergic neuronal differentiation.

If the optimal CHIR99021 concentration for a cell line has been determined in a 2D system, this concentration can be used in this protocol. Alternatively, for more accurate midbrain patterning the CHIR99021 titration can be optimized directly in this protocol, by testing different CHIR99021 concentrations (0.5, 0.6, 0.7, 0.8 μM) in the culture media. The optimal concentration can be determined by the highest percentage of OTX2/EN1 double-positive cells and FOXA2/LMX1A double-positive cells.

## Key resources table

**Table:** 

REAGENT or RESOURCE	SOURCE	IDENTIFIER
**Antibodies**		

Goat anti FOXA2 (1:200)	R&D Systems	Cat#AF2400
Rabbit anti LMX1A (1:5000)	Merck Millipore	Cat#AB10533
Rabbit anti EN1 (1:50)	Sigma	Cat#HPA073141
Goat anti OTX2 (1:500)	R&D Systems	Cat#AF1979
Mouse anti TH (1:2000)	Merck	Cat#MAB318
Rabbit anti TH (1:1000)	Pel-Freez	Cat#P40101-150
Rabbit anti GIRK2 (1:500)	Alomone	Cat#APC-006
Mouse anti-CALB1 (1:5000)	SWANT	Cat#300
Chicken anti-MAP2 (1:2500)	Abcam	Cat#AB92434
Cleaved Caspase-3 antibody (1:400)	Cell Signaling Technology	Cat#9661S
Donkey anti-Goat IgG (H + L) Cross-Adsorbed Secondary Antibody, Alexa Fluor™ 488 (1:1000)	Thermo Fisher Scientific Invitrogen	Cat#A11055
Donkey anti-Rabbit IgG (H + L) Highly Cross-Adsorbed Secondary Antibody, Alexa Fluor™ 568 (1:1000)	Thermo Fisher Scientific Invitrogen	Cat#A10042
Donkey anti-Mouse IgG (H + L) Highly Cross-Adsorbed Secondary Antibody, Alexa Fluor™ 568 (1:1000)	Thermo Fisher Scientific Invitrogen	Cat#A10037
Donkey Anti-Mouse IgG (H + L) Alexa Fluor® 488 AffiniPure (1:200)	Jackson ImmunoResearch	Cat#715-545-150
Donkey Anti-Rabbit IgG (H + L) Alexa Fluor® 488 AffiniPure (1:200)	Jackson ImmunoResearch	Cat#711-545-152
DAPI	Merck Sigma-Aldrich	Cat#D9542

**Chemicals, peptides, and recombinant proteins**		

TeSR E8	STEMCELL Technologies	Cat#05990
Laminin 521 ∗Biolaminin 521 LN (LN521)	BioLamina	Cat#LN521-05
EDTA	Thermo Fisher	Cat#AM9260G
Pluronic F-127	Sigma-Aldrich	Cat#P2443
DMEM/F12	Thermo Fisher	Cat#31331093
Neurobasal media	Gibco	Cat#21103049
Glutamax	Thermo Fisher	Cat#35050038
Pen/Strep	Thermo Fisher	Cat#15140122
PBS ++	Thermo Fisher	Cat#14040091
PBS --	Thermo Fisher	Cat#14190169
N2	Thermo Fisher	Cat#17502048
B27 supplement without Vitamin A	Thermo Fisher	Cat#12587-010
Anti-Adherence Rinsing Solution	STEMCELL Technologies	Cat#07010
Y-27632 ROCK inhibitor	TOCRIS	Cat#1254
SB431542	TOCRIS	Cat#1614
LDN193189	Stemgent	Cat# 04-0074
CHIR99021	Stemgent	Cat#04-0004-10
SAG	Millipore	Cat#566660
FGF8b	R&D Systems	Cat#423-F8
L-Ascorbic acid (AA)	Sigma-Aldrich	Cat#A4403
DAPT	TOCRIS	Cat#2634
Recombinant Human GDNF	R&D Systems	Cat#212-GD-050
LM22A4	TOCRIS	Cat#4607
cAMP	Sigma-Aldrich	Cat#D0627
Paraformaldehyde (PFA)	Sigma-Aldrich	Cat#P6148
Poly(vinyl alcohol) (PVA)	Sigma-Aldrich	Cat#P8136
Glycerol	Sigma-Aldrich	G5516-1L
1,4-Diazabicyclo[2.2.2]octane (DABCO)	Sigma-Aldrich	Cat#D2522
Sucrose	Sigma-Aldrich	Cat#S7903
Triton X-100	Sigma-Aldrich	Cat#X100
O.C.T. Compound	Tissue-Tek	Cat#94-4583

**Experimental models: Cell lines**		

H9		

**Software and algorithms**		

ZEN	ZEISS	
Fuji		
ImageJ		https://imagej.nih.gov/ij/
GraphPad Prism 9.3.0	GraphPad Software, LLC	
CellSens Entry 1.18	Olympus	

**Other**		

35 mm dish	Fisher Scientific	Cat#10170753
AggreWell™400	STEMCELL Technologies	Cat#34411
EB-Disk^360^	Enuvio.com	Cat#eN-eb360u-001
Cell Strainer (40 μm)	Fisher Scientific	Cat#22363547
Superfrost PLUS microscope slides	Thermo Fisher	Cat#J1800AMNZ
Microscope Cover slips	Fisher Scientific	Cat#11911998
Centrifuge	Eppendorf	5804R
Stereomicroscope	Leica	M60
Cryostat NX70	Axlab	Cryostat NX70
Orbital shaker	Thermo Scientific	Cat#88881102

## Materials and equipment

### hPSC culture and ventral midbrain MiCO culture media

#### hPSC culture media

Thaw TeSR™-E8™ 25× Supplement (20 mL) and add into 480 mL of TeSR™-E8™ Basal Medium. Add 2.5 mL Penicillin-Streptomycin (Pen/Strep). Mix thoroughly. Aliquot into 50 mL polypropylene tubes (45 mL/tube) and store at −20°C for up to 6 months. Once the complete TeSR™-E8™ medium is thawed, can be store at 4°C for up to 2 weeks. Do not refreeze thawed media.

***Alternatives:*** Other hPSC culture media such as mTeSR1, mTeSR™ Plus or IPS-Brew Medium can be used.

### Midbrain MiCOs generation and culture media

**Table:** N2B27 media

Reagent	Final concentration	Amount
DMEM/F12 with Glutamax		23.75 mL
Neurobasal medium		23.75 mL
Glutamax (100×)	0.5×	250 μL
Pen/Strep (100×)	0.5×	250 μL
30% Glucose	0.3%	500 μL
ITS-A (100×)	1×	500 μL
N2 supplement (100×)	1×	500 μL
B27 supplement without vitamin A (50×)	0.5×	500 μL
**Total**		**50 mL**

Store up to one month at 4°C.

### Small molecular preparation

#### SB431542

Prepare 50 mM stock in DMSO, aliquot (e.g., in 25 μL) and store at −20°C for up to one year. Prepare 10 mM stock by adding 10 μL of 50 mM SB into 40 μL absolute ethanol and store at −20°C for up to 6 months.

#### LDN193189

Prepare 1 mM stock in DMSO, aliquot (e.g., in 25 μL) and store at −20°C for up to one year. Prepare 100 μM stock by diluted 10 μL of 1 mM stock with 90 μL of absolute ethanol, store at −20°C for up to 6 months.

#### CHIR99021

Prepare 1 mM stock in DMSO, aliquot (e.g., in 25 μL) and store at −20°C for up to one year.

#### SAG

Prepare 200 μM stock in PBS--, aliquot (e.g., in 200 μL) and store at −80°C for up to three years. Thaw aliquot store at 4°C for up to three months.

#### FGF8b

Prepare 25 ng/μL stock in PBS--with 0.1% BSA solution, aliquot (e.g., in 100 μL) and store at −80°C for up to one year. Thaw aliquot store at 4°C for up to one month.

#### LM22A4

Prepare 10 mM stock in H2O, aliquot (e.g., in 50 μL) and stored at −20°C for up to one year. Thaw aliquot store at 4°C for up to three months.

#### L-Ascorbic acid (AA)

Prepare 50 mM stock in H2O, aliquot (e.g., in 500 μL) and stored at −20°C for up to one year. Thaw aliquot store at 4°C for up to three months.

#### GDNF

Prepare 10 μg/mL stock in PBS--with 0.1% BSA solution, aliquot (e.g., in 50 μL) and store at −20°C for up to one year. Thaw aliquot store at 4°C for up to one month.

#### cAMP

Prepare 50 mM stock solution in H2O, aliquot (e.g., in 500 μL) and stored at −20°C for up to one year. Thaw aliquot store at 4°C for up to three months.

#### DAPT

Prepare 10 mM stock in DMSO, aliquot (e.g., in 50 μL) and store at −20°C for up to one year. Prepare 1 mM stock by diluted 10 μL of 10 mM stock with 90 μL of absolute ethanol, store at −20°C for up to 6 months.

#### Y-27632 ROCK inhibitor

Prepare 50 mM stock solution in H20, aliquot (e.g., in 20 μL) and store at −20°C for up to one year. Prepare 10 mM stock solution by diluted 10 μL of 50 mM stock with 40 μL of DMEM, store at 4°C for up to three months.

**Table:** Midbrain floor plate patterning media (day0-day9)

Reagent	Final concentration	Amount
N2B27 media		10 mL
SB (10 mM)	10 μM	10 μL
LDN (100 μM)	100 nM	10 μL
CHIR (1 mM)	0.6 μM (for H9 cell line)	6 μL
SAG (200 μM)	400 nM	20 μL
**Total**		**10 mL**

Small molecules are added fresh every time.

**Table:** Midbrain floor plate patterning media (day 9-day 11)

Reagent	Final concentration	Amount
N2B27 media		10 mL
FGF8b (25 ng/μL)	100 ng/mL	40 μL
**Total**		**10 mL**

Small molecules are added fresh every time.

**Table:** Midbrain floor plate patterning media (day 11-day 16)

Reagent	Final concentration	Amount
N2B27 media		10 mL
FGF8b (25 ng/μL)	100 ng/mL	40 μL
LM22A4 (10mM)	2 μM	2 μL
AA (50 mM)	200 μM	40 μL
**Total**		**10 mL**

Small molecules are added fresh every time.

**Table:** NDM Media

Reagent	Final concentration	Amount
Neurobasal medium		48.25 mL
B27 supplement without vitamin A (50×)	1×	1 mL
Glutamax (100×)	1×	500 μL
Pen/Strep (100×)	0.5×	250 μL
**Total**		**50 mL**

Store up to two weeks at 4°C.

**Table:** Maturation media (From day 16)

Reagent	Final concentration	Amount
NDM media		10 mL
LM22A4 (10 mM)	2 μM	2 μL
AA (50 mM)	200 μM	40 μL
GDNF (10 μg/mL)	10 ng/mL	10 μL
dcAMP (50 mM)	500 μM	100 μL
DAPT (1 mM)	1 μM	10 μL
**Total**		**10 mL**

Small molecules are added fresh every time.

### Histology

#### Fixation solution

Prepare 4% PFA by thaw 1 mL of 16% PFA (from −20°C), add 3 mL of PBS (pH 7.4). 4% PFA can be stored at 4°C for up to one month. Prepare 16% PFA by adding 8 g PFA powder into 35 mL H2O, heat up to 60°C (do not reach higher than 60°C). Add 10 M NaOH until PFA just dissolves, add 5 mL 10× PBS. When the solution cools down to room temperature (20°C–25°C), use pH strips to check the pH and add NaOH or HCl to adjust the pH to 7.4. Add dH2O to reach 50 mL solution. Aliquot and store at −20°C for up to 6 months.

#### Blocking solution for immunostaining

Prepare 5% donkey serum in PBS with 0.25% triton-X solution.

#### PVA-DABCO mounting solution

Prepare 50 mL of 5% PVA-DABCO solution by adding 4.8 g of PVA into 12 g of glycerol. Mix well until PVA disperse well in the glycerol solution, then add 12 mL distilled water, mix overnight (16–20 h) at room temperature (20°C–25°C). Add 24 mL of 0.2 M Tris-HCL at pH 8–8.5. Heat the solution to 50°C in a water bath with mixing for approx. 30 min. Then add 2.5 g DABCO. Centrifuge at 5000 x g for 15 min. Remove supernatant, aliquot (e.g., in 1 mL), store at −20°C for up to 6 months and up to one week at 4°C. Do not refreeze.

**CRITICAL:** PFA and DABCO are hazardous. Wear gloves and use only in a fume hood.

## Step-by-step method details

### Maintain and passage hPSCs (Day −5)

#### Coating the dish(es) for hPSCs culture

**Timing: 2–16 h**

1. Resuspend a frozen aliquot of BioLamina 521 (LN521, 100 μg/mL) in 1 mL PBS++ to have a final concentration of 0.5 μg/mL LN521 (50 μL LN521 per mL PBS++).
  a. Mix well without vortexing.
  b. Add 1 mL to coat the dish (Ø35 mm dish).
2. Leave the coated dish at 37°C incubator for 2 h or at 4°C overnight (16–20 h).

***Note:*** The coated dish(es) can be kept at 4°C for up to 4 weeks, make sure the coated surface does not dehydrate. LN521 coating solution can not be reused.

#### Passage hPSCs

**Timing: 30 min**

This step is to grow hPSCs to 75%–80% confluency for starting a midbrain MiCO experiment. hPSCs are cultured under feeder-free condition on Biolaminin (LN521, 0.5 μg/cm^2^ in PBS++) coated dishes. We used gentle cell dissociation buffer (0.5 mM EDTA in PBS- -) to passage the hPSCs and plated 40,000–50,000 cells on a dish (Ø = 35 mm, 9 cm^2^) and cultured for 5 days. We normally start this step on a Wednesday, and then the cells will be ready to be used on a Monday to start a vmDA MiCOs generation experiment.

3. Gentle cell dissociation buffer is prepared by adding 10 μL of EDTA (0.5 M) to 10 mL of PBS- - to have final 0.5 mM EDTA solution.
4. Pre-warm E8 media to room temperature (20°C–25°C).
5. Aspirate LN521 solution from the pre-coated dish(es) and replace with 1 mL of fresh E8 media.
6. Aspirate the hPSCs culture media and wash the cells one time with PBS--.
7. Aspirate the PBS--, and add 1 mL of gentle cell dissociation buffer.
  a. Wait for 6–8 min at room temperature (20°C–25°C).

***Note:*** The incubation time can vary between different cell lines. It also depends on how recently the gentle cell dissociation buffer was prepared. We recommend using the gentle cell dissociation buffer within two weeks.

8. Aspirate the gentle cell dissociation buffer, add 1 mL fresh E8 media.
  a. Use P1000 pipette to scrape cells off from the dish.
  b. Pipette the cell suspension once to twice to break the colonies into smaller pieces.
  c. Collect the cells and transfer them to a 1.5 mL Eppendorf tube.
9. Use a P200 pipette to take out 100 μL cell suspension and transfer to a new 1.5 mL Eppendorf tube.
  a. Pipette up and down about 20–25 times to have single cell suspension.
  b. Take out 10 μL for counting the cell density.
10. Calculate cell number and plate 40,000 cells on the pre-coated LN521 dish with E8 media. Make sure the cells distribute uniformly on the dish(es).
11. Transfer the dish(es) into a 37°C incubator. Media change every two or three days.

***Note:*** Cell seeding density is cell line dependent. For GBA-002-C3 (DANi-002C) cell line, we need to seed 8000 cells/cm^2^.

### Start a midbrain MiCO generation experiment (Day 0)

**Timing: 1.5 h**

This step is to generate MiCOs from AggreWell™400 24-well plate. We have tested cell numbers ranging from 50 to 1000 per MiCO. And we recommend that 300 to 750 cells per MiCO is a good range for generating MiCOs.

### Prepare AggreWell**™**400 24-well plate

12. Add 500 μL Anti-Adherence Rinsing Solution to the number of wells to be used.
13. Centrifuge 1300 x g for 5 min.
14. Check the microwells under a microscope to ensure that there are no bubbles in wells. If there are any bubbles, repeat step 13.
15. Aspirate Anti-Adherence Rising Solution from the well(s).
16. Rinse the well(s) with pre-warm N2B27 media (2 mL/well).
17. Aspirate N2B27 media and add prewarm N2B27 media (1 mL/well) containing small molecules (Midbrain floor plate patterning media (day0-day9)) and 10 μM ROCK inhibitor.
18. Leave the AggreWell™400 24-well plate in the incubator at 37°C.

***Note:*** A balance plate is needed for step 13.

### Prepare hPSCs

19. Under the stereomicroscope, use a glass pipette to scrape off and remove any differentiated cells.
20. Aspirate E8 medium from the dish.
21. Gently wash the cells with 1 mL PBS--.
22. Aspirate the PBS--, add 1 mL of Gentle Cell Dissociation Buffer.
  a. Wait for 6–8 min at room temperature (20°C–25°C).
23. Aspirate the gentle cell dissociation buffer, add 1 mL N2B27 containing Small Molecules (Midbrain floor plate patterning media (day0-day9)) and ROCK inhibitor.
24. Use P1000 pipette to scrape cells off from the dish.
  a. Pipette the cell suspension once to twice to break the colonies into smaller pieces.
  b. Collect the cells and transfer them to a 1.5 mL Eppendorf tube.

### Seeding hPSCs on AggreWell**™**400 24-well plate

25. Use a P200 pipette to take out 100 μL cell suspension from step 24 and transfer to a new 1.5 mL Eppendorf tube.
  a. Pipette up and down about 20–25 times to have single cell suspension.
  b. Take out 10 μL for counting the cell density.
  c. Repeat cell counting and aim for the difference between the two counts is less than 20%.
26. Calculate the volume of cell suspension to be used for seeding. Add the cell suspension to the well of Aggrewell™400 and add N2B27 containing Small Molecules (Midbrain floor plate patterning media (day0-day9)) and 10 μM ROCK inhibitor to achieve a final volume of 2 mL/well.

***Note:*** For step 26, the cell suspension volume added should be less than 1 mL. If the cell density is too low, spin down and resuspend to have cell density above 1 million cells/mL. There are approximately 1,200 microwells per well of the AggreWell™400 24-well plate. If desired 500 cells/microwell then seed 600,000 (500∗1200) live cells per well (troubleshooting 1).

For a new cell line, cell number titration can be done by seeding different cell amounts per MiCO. For example, if the cell numbers per MiCO are 300, 500, and 750, three wells of AggreWell™400 will be seeded with 360,000 (300∗1200), 600,000 (500∗1200), and 900,000 (750∗1200) live cells, respectively. MiCOs are maintained as described below and sizes of the MiCOs will be measured and necrotic center formation will be tested by Cleaved Caspase-3 staining. The optimized cell amount is achieved when the size of the MiCOs do not exceed 800 μm and no necrotic center is detected.

27. Pipette cells carefully up and down several times without introducing bubbles to the well.
28. Immediately centrifuge at 100 x g for 3 min and check even cell distribution under a microscope.
29. Place the plate in the incubator. This day is day 0 of the culturing period.

***Note:*** For step 28, a balance plate is needed.

### Half-medium change for AggreWell**™**400 24-well plate (Day 2- Day9)

**Timing: 10 min**

30. Half media change with P1000 every second day (or more often if medium color changes to yellow) by slowly remove 1 mL media from the top of the liquid and add 1 mL of new media without disturbing the MiCOs.
31. Use Olympus IX53 microscope to follow the size of the MiCOs. On day 4 to day 9 when the MiCO size reaches approximately 200 μm following the step 40- step 49, transfer the MiCOs to EB-Disk^360^.

### Transferring from AggreWell**™**400 to EB-Disk^360^

#### Coating EB-Disk^360^ for MiCO culture

**Timing: 24 h**

32. Under aseptic conditions add 3 mL of 5% Pluronic F-127 solution per well to the 6-well plate.
33. Place the EB-Disk^360^ in the well using a sterile tweezer. While placing, tilt the EB-Disk^360^ a little to avoid bubbles underneath the disk.
34. Centrifuge at 300 x g for 1 min.
35. Seal the 6-well plate with parafilm and keep at 4°C for 12–16 h.
36. Aspirate 5% Pluronic F-127 solution.
37. Rinse the EB-Disk^360^ with 3 mL PBS --.
38. Rinse the EB-Disk^360^ with 3 mL N2B27.
39. Aspirate and add 3 mL complete medium per well.

***Note:*** The coating solution 5% Pluronic F-127 for step 35 can be stored for at least 8 months at 4°C. The EB-Disk^360^ will not stay at the bottom of the well but will be floating in the medium.

For step 34, a balance plate is needed.

### Harvest MiCOs from AggreWell**™**400 and transfer to EB-Disk^360^

**Timing: 45–60 min/well**

40. Place an inverted 40 μm cell strainer on top of a 50 mL tube.
41. Use a 2 mL serological pipette to carefully dislodge the MiCOs from the AggreWell™400 and transfer to the inverted 40 μm cell strainer (troubleshooting 2).
42. Use room temperature (20°C–25°C) PBS++ to rinse the AggreWell™400 to get all of the MiCOs onto the cell strainer.
43. Carefully rinse the cell strainer with the MiCOs with PBS++.
44. Invert the cell strainer to its correct position and place it in a clean dish (Ø = 35 mm).
45. Elute the MiCOs from the cell strainer with 2 mL complete medium in the dish.

***Note:*** Within 1–2 hours, the MiCOs will start sticking together. Therefore, it is preferred only to harvest from one well of AggreWell™400 at a time (troubleshooting 3).

46. Move the MiCOs with p200 to pre-coated EB-Disk^360^.
47. Under microscope carefully distribute the MiCOs in the wells of the EB-Disk^360^.

***Note:*** The EB-Disk^360^ contains 360 microwells with the size 900 μm. Therefore, one EB-Disk^360^ can hold 360 MiCOs.

48. Leave on an orbital shaker at 70 rpm in the incubator.
49. Change half medium every 3–4 day (or more often if medium color changes to yellow) by slowly remove 2 mL old media and add 2–3 mL fresh media.

### Collecting MiCOs for analysis

**Timing: 10 min**

50. Place the EB-Disk^360^ under Stereomicroscope in the flow bench, use a P1000 pipette to take out MiCOs from the microwell. This can be done at different timepoint and for different assays.

***Note:*** We have performed analysis such as immunostaining, HPLC, RNA and protein extraction on the MiCOs.

### Fixation and embedding MiCOs in O.C.T. Compound

**Timing: 2 days**

51. MiCOs are collected in a 1.5 mL Eppendorf tube or a center well plate, wash with PBS- -.
52. Remove PBS- -, add 500 μL 4% PFA and leave for 20 min at room temperature (20°C–25°C) (troubleshooting 4).
53. Remove 4% PFA and wash 3 times with PBS--, 10 min each time.
54. Remove PBS--, add 20% sucrose without disturbing the MiCOs, leave overnight (16–20 h) at 4°C.
55. When the MiCOs sink to the bottom of the Eppendorf tube (or the center well plate), pour them to a center well plate.
56. Use tweezers and transfer the MiCOs to a plastic container with O.C.T. Compound.
57. Use the tweeze to move the MiCOs to the bottom and center part of the embedding container, be careful not crush the MiCOs.
58. Transfer the embedded MiCO block to a 96% EtOH dry ice bath until the block change color from transparent to a white color.
59. Label the samples and store them at −80°C until sectioning with Cryostat NX70.
60. Use a Cryostat NX70 to section the MiCOs with a thickness of 10 μm. Collect 3–4 sections on a glass slide. Store the slides at −80°C until immunostaining is to be performed.

***Note:*** PFA is hazardous. Wear gloves and use in a fume hood.

When samples are taken out from −80°C for sectioning, it is recommended to wait for 20–30 min until the samples reach a temperature of −20°C.

### Immunostaining of midbrain MiCO sections

**Timing: 2 days**

61. The slides are permeabilized with PBT (0.25% Triton X in PBS) for 10 min at room temperature (20°C–25°C).
62. Remove PBT. Incubate with blocking solution (5% donkey serum in PBT) for 1 h at room temperature (20°C–25°C).
63. Remove blocking solution. Use a PAP pen to draw a circle around the MiCO sections, apply the primary antibodies in blocking solution and incubate at 4°C overnight (16–20 h).
64. Remove primary antibodies and wash 3 × 10 min with PBT.
65. Block for 10 min with blocking solution.
66. Remove blocking solution. Incubate with secondary antibodies diluted in blocking solution for 1 h at room temperature (20°C–25°C) in a humid chamber in a dark environment.
67. Remove secondary antibodies. Wash 3 × 10 min with PBT.
68. Remove PBT. Incubate with 2-(4-Amidinophenyl)-6-indolecarbamidine dihydrochloride (DAPI) diluted in PBS for 5 min.
69. Remove DAPI. Wash 3 × 5 min with PBS.
70. Remove PBS. Mount the slides with coverslips using DABCO.
71. Leave the slides to dry under a fume hood in dark. Store the stained slides at 4°C protected from light.
72. Use a microscope (Zeiss LSM780 confocal microscope) to take images.

## Expected outcomes

**Figure 1:**
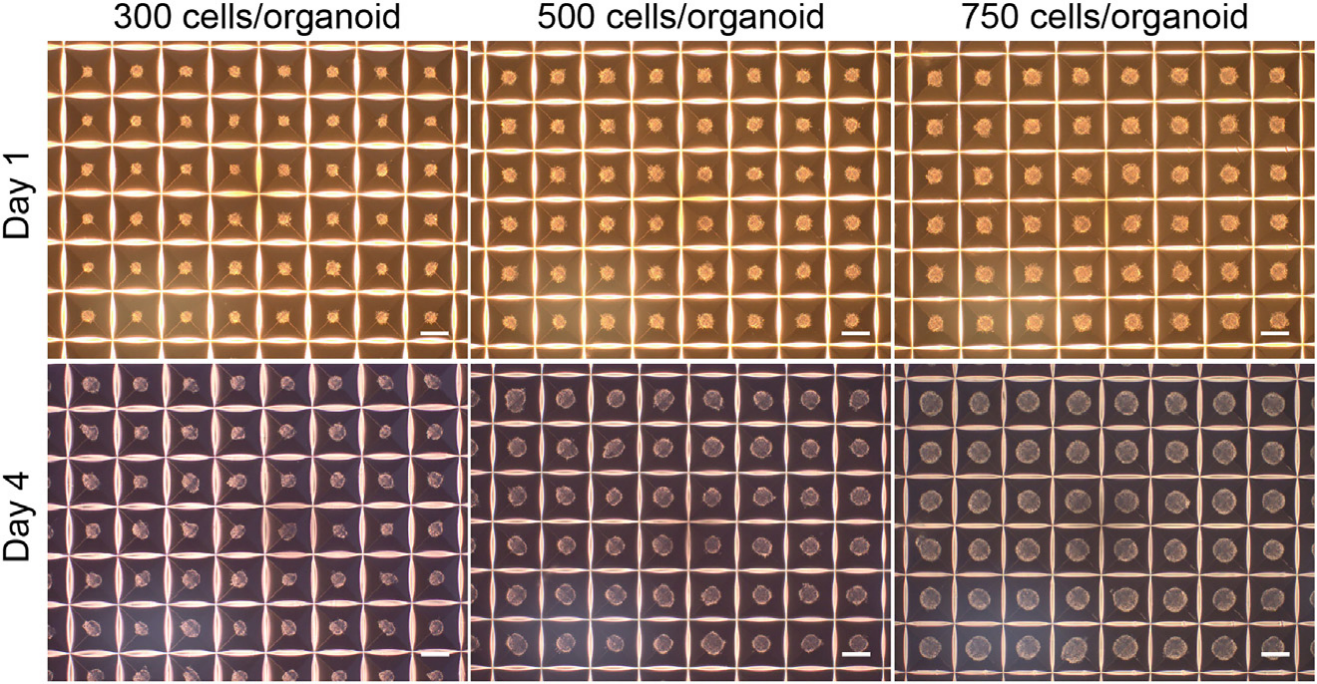
Generation of ventral midbrain miniaturized human organoids (MiCOs) from hPSCs in AggreWells^TM^ Representative pictures of human vmDA organoids still within AggreWells^TM^ from day 1 and day 4. Cell number per MiCO at seeding was 300, 500, and 750. Scale bars, 100 μm.

**Figure 2:**
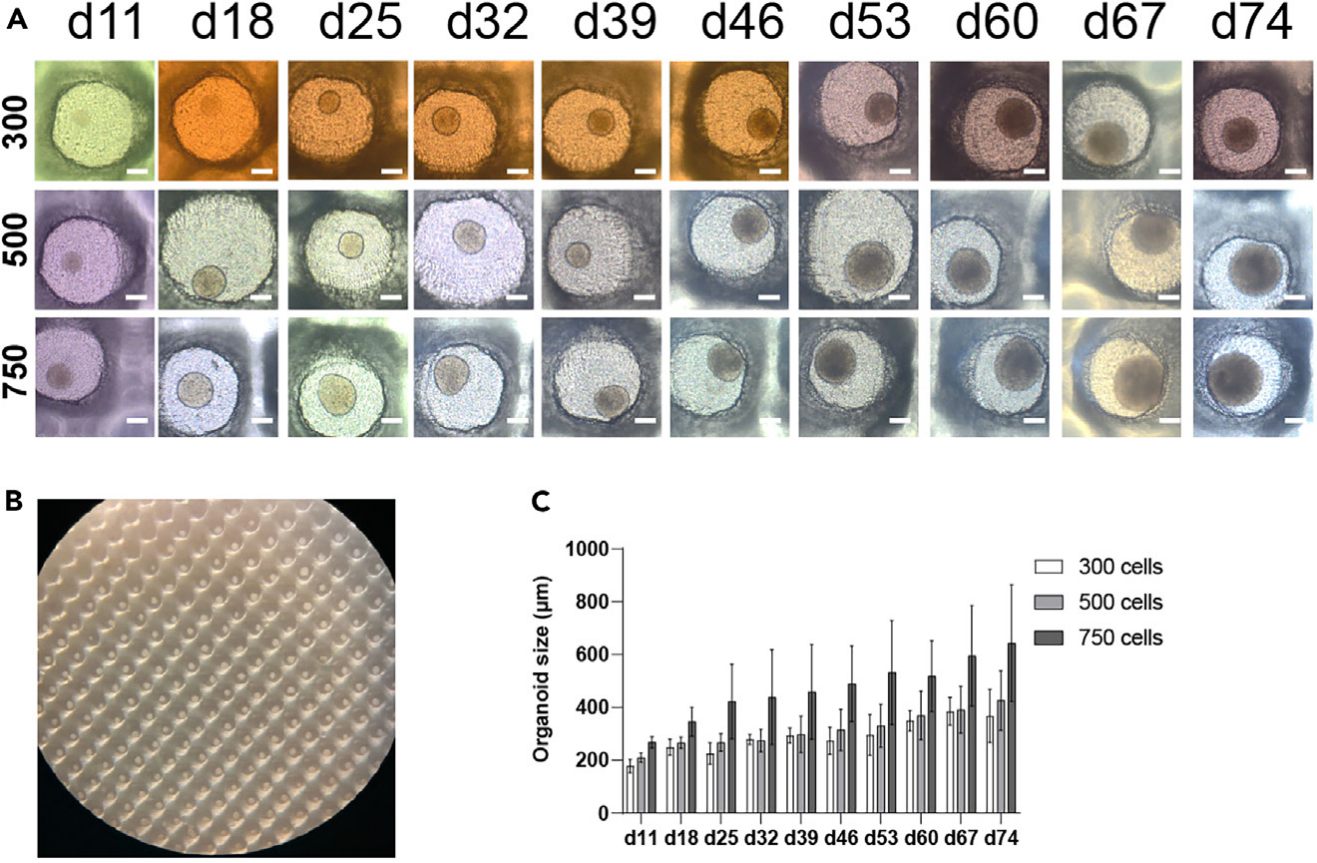
Long-term maintain miniaturized human vmDA MiCOs in EB-Disk^360^ cultureware (A) Representative pictures of human vmDA MiCOs (with start cell numbers of 300, 500, and 750 cells per MiCO) in the EB-Disk^360^ up to day 75. Scale bars, 100 μm. (B) Overview of an EB-Disk^360^ with human vmDA organoids. (C) The size of organoids during *in vitro* maintenance. The data are presented as the mean ± SD; n = 6-18, from 3 independent experiment setups.

Reproducible MiCOs can be maintained with this protocol for up to 150 days. This protocol is an efficient and cost-effect way to maintain and change media for up to 360 MiCOs in one well of a 6-well plate. As shown in Figure 1, 1200 MiCOs can be generated in one well of 24-well plate format in the AggreWell^TM^400. MiCOs can be transferred to EB-Disk^360^ from day 4 to day 9. From then on, the MiCOs are maintained in the EB-Disk^360^ for up to 5 months. The MiCOs remain intact and grow slowly during further differentiation and maturation (Figure 2) which is key to avoiding the formation of a necrotic core.

**Figure 3:**
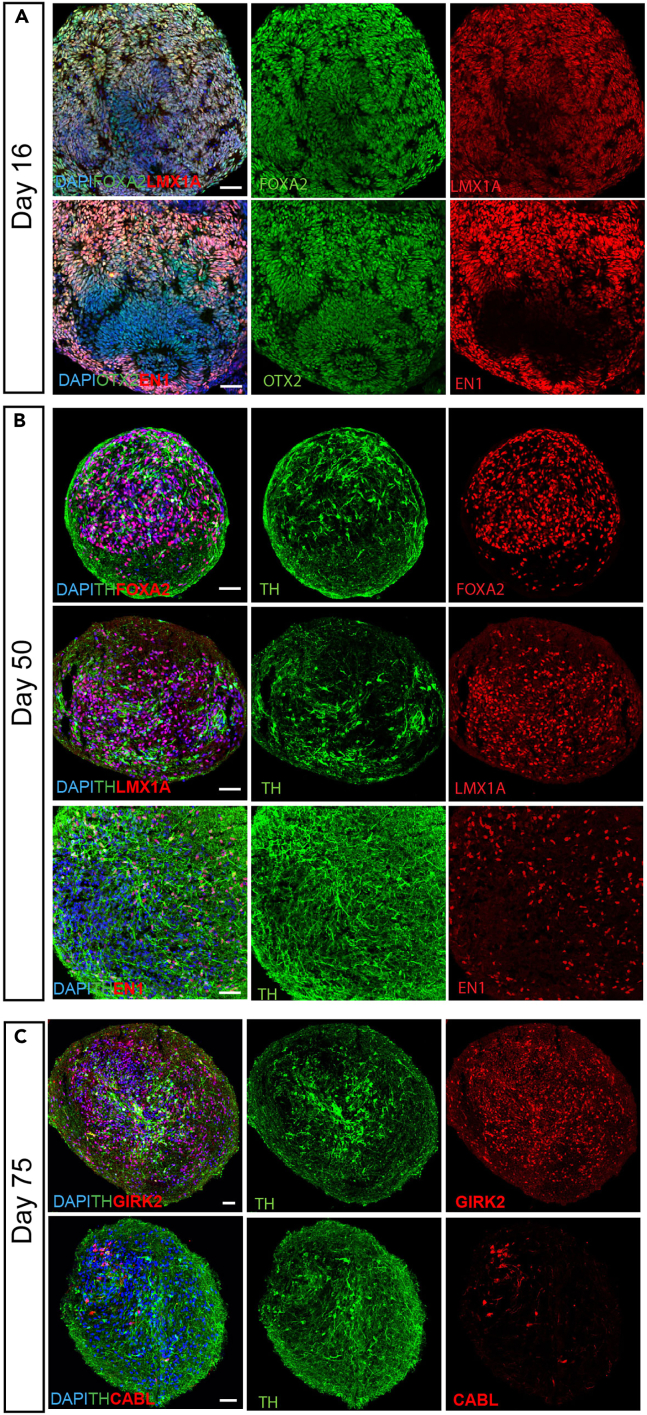
Immunostaining of miniaturized human vmDA MiCOs (A) Representative images of immunostaining of FOXA2/LMX1A and OTX2/EN1 in human vmDA MiCOs at day 16. Scale bars, 50 μm. (B) Representative images of immunostaining of FOXA2/TH, LMX1A/TH, and EN1/TH in human vmDA MiCOs at day 50. Scale bars, 50 μm. (C) Representative images of immunostaining of GIRK2/TH and CALB/TH in human vmDA MiCOs at day 75. Scale bars ,50 μm.

**Figure 4:**
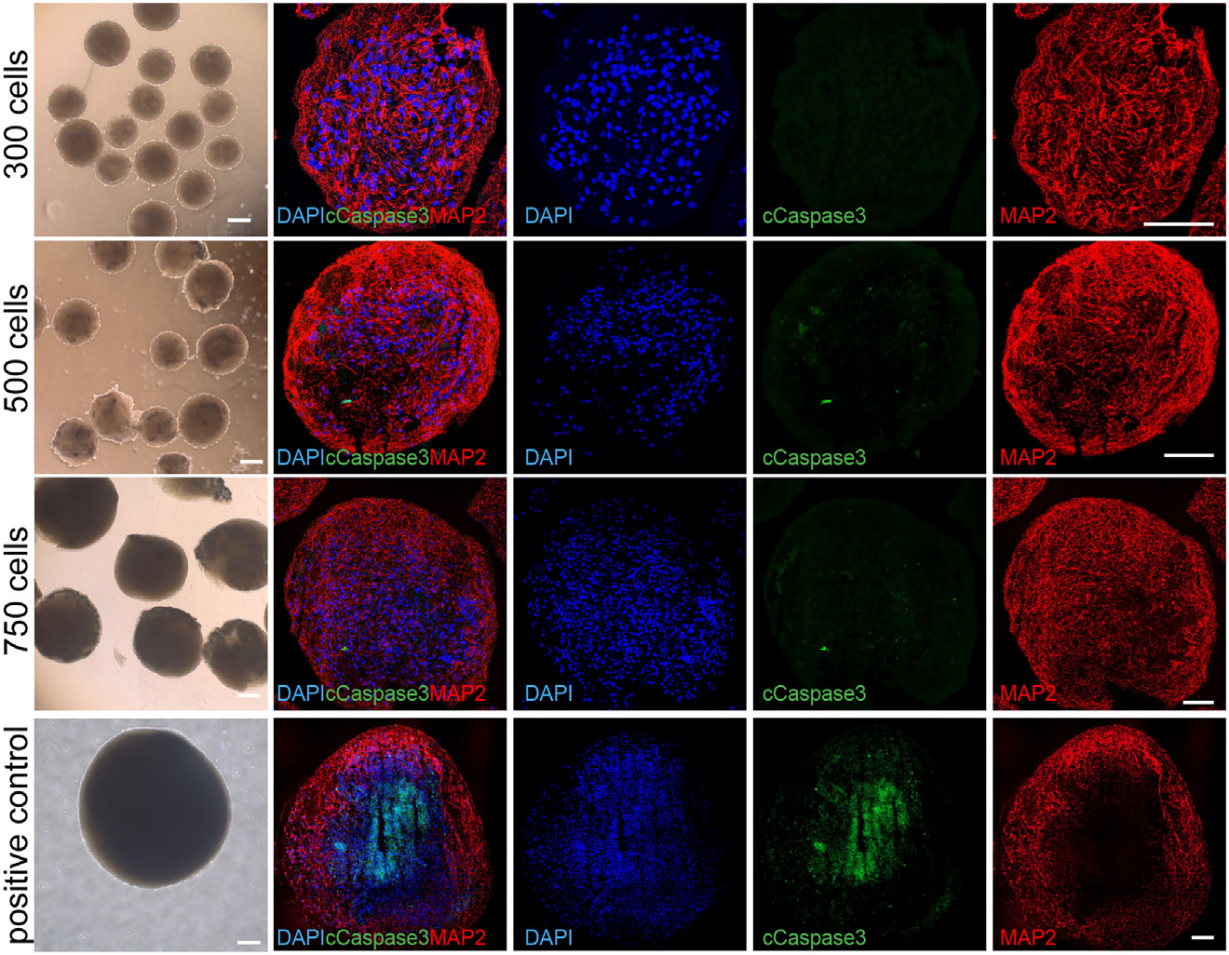
Immunostaining of Cleaved Caspase 3 (cCaspse 3) co-stained with MAP2 in miniaturized human vmDA MiCOs at day 75 Scale bars, 100 μm. Positive control for cCapase3 was from an organoid with diameter around 1 mm.

vmDA MiCOs will express floor plate progenitor markers such as LMX1A/FOXA2, as well as caudal midbrain makers OTX2/EN1 on day 16.^2^^,^^3^^,^^4^^,^^5^ To confirm the presence of vm dopaminergic neurons, immunostaining with TH and co-staining with FOXA2, LMX1A, and EN1 was performed and showed robust expression at day 50^4,5^. On day 75, majority of TH cells were positive with GIRK2 staining while a small portion of TH cells were positive with CALB staining (Figure 3), which indicating that the protocol generate more A9 neurons compared to A10 neurons.^6^^,^^7^ TH and MAP2 staining are seen both on the edge and the center of the MiCOs (Figures 3 and 4). The size of the MiCOs did not exceeded 800 μm and most remarkably there was no necrotic center detected at day 75 (Figure 4) (troubleshooting 5).

***Note:*** It is suggested to do immunostainings on day 16 to make sure the progenitors are patterned correctly (positive staining for FOXA2/LMX1A and OTX2/EN1) before extending the MiCOs for long-term culture. In case of the cell patterning is wrong, stop the experiment and start a new round.

Depending on the downstream analysis, MiCOs can be taken out for different assays such as western blot, dopamine measurement, electrophysiology and so on. For example, MiCOs can be cultured individual in a multi-well plate such as a 96-well plate for drug screening.

## Limitations

This protocol requires the titration of the CHIR concentration for each individual cell line to ensure high efficiency of ventral midbrain NPCs and dopaminergic neurons.

Depending on the experimental set up and downstream analysis, long-term maintenance of the MiCOs are required, which can take months.

It is time consuming when transferring MiCOs from AggreWell™400 to EB-Disk^360^ (30–45 min for transferring 360 MiCOs into an EB-Disk^360^). However, once this step is done, it is very easy to change media and culture/maintain the MiCOs in this format. This format also saves media compared to using a spinner flask culture system and avoids using Matrigel to embed the MiCOs.

In the MiCO system, we have not looked for the presence of alpha-Synuclein protein aggregates or disease related mechanisms. However, patient-derived human iPSCs differentiated into mesencephalic dopaminergic neurons in organoids have shown evidence of aggregated synuclein and progressive loss of dopaminergic neurons.^8^^,^^9^

## Troubleshooting

### Problem 1

Cell number per MiCO is less than 50 or higher than 1000 from a healthy cell line will be difficult to use this protocol.

### Potential solution

**Figure 5:**
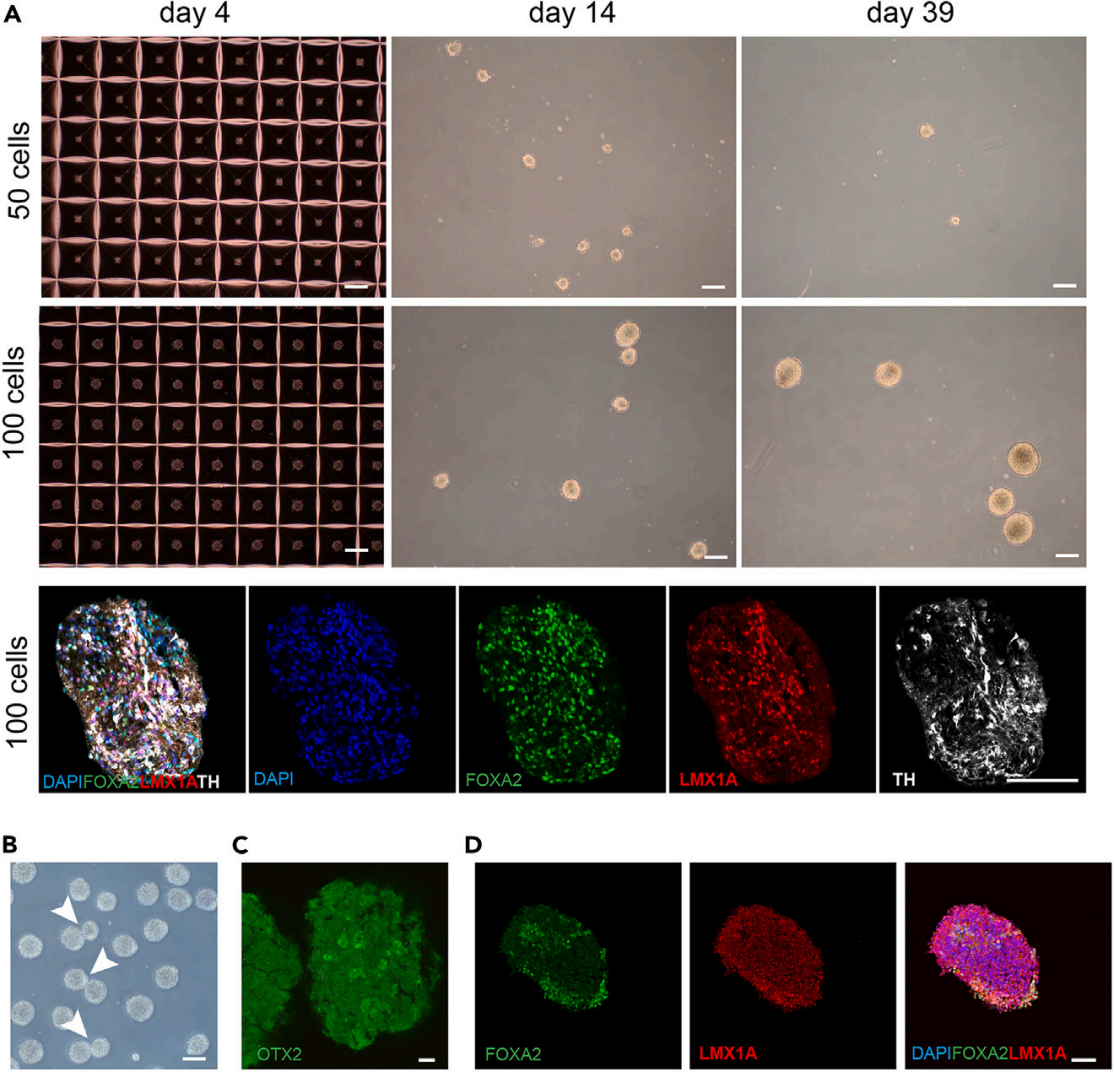
Troubleshooting (A) Cell number per MiCO at seeding should be higher than 100 cells on day 0. When the number is 50 cells per MiCO, it is difficult to maintain the MiCOs and challenge for immunostaining analysis. We succeed in maintaining and immunostaining for the MiCOs generated as 100 cells per MiCO at seedingand culture up to day 42. Scale bars, 100 μm. (B) Some MiCOs are sticking together (pointed with arrowhead) when they stayed in a dish for longer than 1–2 h. Scale bars, 100 μm. (C) Fail immunostaining when organoid fix for 2 h at room temperature (20°C–25°C). Scale bars, 10 μm. (D) Few positive cells stained with FOXA2/LMX1A at day 16. Scale bars, 50 μm.

Cell number per MiCO is optimized to be between 100 to 750 for a non-disease cell line. We have tested lowest cell numbers per MiCO from 50 cells and 100 cells per MiCO. When the cell number is 50 per MiCO, the MiCO is difficult to maintain long-term and challenging to analyze. The lowest cell number per MiCO we tested is 100 cells, and we succeed with maintain these until day 42. (Figure 5A).

For disease cell lines, cell number titration needs to be tested.

### Problem 2

There is a risk that MiCOs may break or stick onto the cell strainer during dislodgment and transfer.

### Potential solution

Slowly pipette up and down 3–5 times with the 2 mL serological pipette in the AggreWell so that most of the MiCOs are transfered from the AggreWell™400 to the inverted 40 μm cell strainer. Then quickly use PBS++ to rinse the AggreWell™400 to get all of the MiCOs onto the cell strainer and rinse again with PBS++ to avoid MiCOs dehydrated and stick onto the cell strainer. And finally, quickly elute the MiCOs from the cell strainer (need to invert the cell strainer to make sure it is in the correct position) with 2 mL complete media into a clean dish.

### Problem 3

MiCOs will stick together when harvesting them in a dish for longer than 1–2 h at room temperature (20°C–25°C) (Figure 5B).

### Potential solution

It is important to harvest one well of an AggreWellTM 400 and transfer the MiCOs to EB-disk360 at a time. If it takes longer than 1 h to transfer, we recommend using 10 mL complete medium in the dish (Ø = 100mm) to help keep the MiCOs separated.

### Problem 4

Poor immunostaining result from day 16 samples (MiCOs generated from 100 cells per MiCO on day 0) when 4% PFA fix for 2 h at room temperature (20°C–25°C) (Figure 5C).

### Potential solution

Fixation time needs to be optimized. Since the MiCOs generated from this protocol are less than 1 mm in size, the 4% PFA fixation time needs to be shorten to 20 min at room temperature (20°C–25°C). For other antibodies not mentioned in this protocol, fixation time may need to be optimized.

### Problem 5

MiCOs did not show correct patterning with fewer cells positive immunostainings with FOXA2/LMX1A on day 16.

### Potential solution

Start new experiment round with new small molecular aliquots.

## Resource availability

### Lead contact

Further information and requests for resources and reagents should be directed to and will be fulfilled by the lead contact, Mark Denham (mden@dandrite.au.dk).

### Materials availability

Not applicable.

## Data Availability

Not applicable.
